# Evidence for a maximum “shelf-life” of oocytes in mammals suggests that human menopause may be an implication of meiotic arrest

**DOI:** 10.1038/s41598-018-32502-2

**Published:** 2018-09-20

**Authors:** Susanne Huber, Martin Fieder

**Affiliations:** Department of Anthropology, University of Vienna, Vienna, Austria

## Abstract

There is an ongoing debate why a trait like human menopause should have evolved. Adaptive explanations explain menopause with fitness benefits of ceasing reproduction, whereas non-adaptive explanations view it as an epiphenomenon. Here we present data in support of non-adaptive explanations of menopause suggesting a maximum shelf-life of oocytes. By analyzing the association between lifespan and age at reproductive senescence across 49 mammal species, we find that the positive association levels off in long lived species, indicating that the age at reproductive senescence has an upper limit. Only in baleen whales there seems to be no evidence for reproductive senescence. We suggest that apart from the baleen whales, the confinement of reproductive senescence in long-lived species may be the result of physiological constraints imposed by the long period of time oocytes remain inactive in an arrested phase of meiosis from their production *in utero* until ovulation. We therefore conclude that menopause may be an implication of the long duration of meiotic arrest caused by semelgametogenesis together with long lifespan.

## Introduction

Human menopause is the irreversible cessation of menstrual cycling that typically occurs at the age of 45 to 55 years^1^, leaving about 30 years of post-reproductive life-span. From an evolutionary perspective, it is not clear, why a trait like menopause should have evolved. As evolution favors increased reproduction, there should not be selection for post-reproductive individuals^2^. Generally, evolutionary biologists have considered two main types of explanations of menopause^3,4^: adaptive hypotheses, stating that menopause itself has been positively selected for, and non-adaptive hypotheses, assuming that menopause is an epi-phenomenon that has not been directly selected for. Both types of explanation, however, are not necessarily mutual exclusive. According to Nichols *et al*.^5^, prolonged post-reproductive stage may be a consequence of non-adaptive origins followed by adaptive evolutionary ‘tinkering.

Adaptive explanations of menopause assume that by ceasing reproduction, females gain fitness benefits. The two main hypotheses are the “mother”- and the “grandmother”–hypotheses, considering menopause as an adaptation facilitating increased investment in already existing offspring^6–9^. The mother hypothesis states that cessation of reproduction to invest in living offspring may be evolutionarily advantageous if offspring survival depends on maternal care and mortality risk due to childbirth increases with age^2,6,9–13^. The grandmother hypothesis assumes that older females increase their inclusive fitness by investing in their grandchildren’s survival rather than giving birth to own children^14–18^. Evidence, however, is equivocal^3,8,13,14,19,20^. The very recent finding of lower average offspring number in women living with their mother or mother-in-law in the household (on the basis of over 2 million data from 14 countries across the world) also provides no support^21^. This lack of conclusive empirical evidence for adaptive explanations gives reason to re-consider non-adaptive explanations.

Non-adaptive hypotheses explain menopause as an epi-phenomenon of selection for efficient reproduction early in life at the expense of reproductive senescence^22,23^, or as a by-product of an increase in life expectancy^24,25^. These hypotheses act on the assumption that evolution is constrained by phylogenetic, developmental, and genetic constraints^26^ such as antagonistic pleiotropy causing increased fertility in early life but aging in later life^2^, or physiological constraints preventing that increases in longevity are accompanied by a prolongation of fertility. Here, exhausting viable egg supply resulting in a depletion of viable egg stores is considered the crucial physiological constraint causing reproductive senescence in female mammals^27,28^ although according to vom Saal *et al*.^29^ the view that oocyte exhaustion is the only cause of reproductive senescence in mammals is too simplistic.

Male mammals usually also show some reproductive decline with age but they remain capable to reproduce virtually until death^29^. A fundamental difference between male and female mammals is that in contrast to males, females are not able to continue to produce oocytes past their intrauterine phase (‘semelgametogenesis’)^29^, but see^30^. Hence, female mammals are born with a finite stock of oocytes^29^, whereas in male mammals, spermatozoa develop continuously during their reproductive lifespan^31^. In women, all gametes are developed by the fifth month of gestation and approximately 400 oocytes are ovulated during lifetime^23^. Menopause occurs when this stock of oocytes is depleted so as to be no longer sufficient to support ovulation^29^. Given that until the fifth month of pregnancy up to 7 million germ cells are produced that decline to 1–2 million follicles at birth and about 400000 at puberty, with still 1,000–10,000 follicles remaining at the age 40^4,32^, according to Faddy *et al*.^33^, human females should have enough oocytes to last 70 years, even though menopause occurs at approximately 50 years of age.

So, it may not only be the number of oocytes or the rate of atresia that constrains the fertile period but the time span between germ cell development and ovulation itself. Accordingly, it has been suggested that the length of time oocytes can remain viable may be the limiting factor of reproductive lifespan, assuming that this limitation of reproductive lifespan would constrain reproduction in female mammals whose lifespan exceeded such a putative maximum shelf-life of oocytes^34,35^.

The present study was aimed at testing the assumption of a maximum shelf-life of oocytes by analyzing the association between maximum lifespan and the age of reproductive senescence (proxied by the age at last reproduction or, in humans and two non-human primates, the age at menopause) across 49 mammal species. In case a maximum shelf-life of oocytes existed, we would expect that in long-lived species, age at reproductive senescence should have an upper limit independent of maximum lifespan. Even though the question whether mammals species exhibit post-reproductive lifespans has been widely discussed, suggesting it either a common trait^5,28,36^, or limited to humans and few toothed whales^34,37,38^, to our knowledge, the association between lifespan and age at reproductive cessation is so far unexamined.

## Methods

We used data provided by the database AnAge^39^ for our analysis, including each mammal species for which data on maximum lifespan (LS) and age at reproductive senescence (RS) was provided (26 species). Maximum lifespan is estimated by AnAge from record longevity; however, anecdotes have not been used to estimate maximum lifespan. For age at reproductive senescence, we also used the maximum value. In order to expand the number of long-lived species, we further performed a literature search in Google scholar on all species listed in the AnAge database with a maximum lifespan of 50 years and older and included each species in the analysis, for which we found data on age of reproductive senescence (11 species). In addition, we included data provided by Cohen^36^ (9 species) and vom Saal *et al*.^29^ (3 species) in case accurate data were presented, in the analysis. We used data obtained both from captive and wild animals. Also, the quality of the data greatly varies among species. The mixing of data from wild and captive animals may rather generate a type II than a type I error because in the long-lived species, data obtained from wild animals are overrepresented. In wild animals, in turn, maximum lifespan may be undervalued compared to data from captive animals, which are protected from extrinsic factors such as predation. In the narwhal, for instance, according to Garde *et al*.^40^ maximum age may be underestimated because it is obtained from wild individuals of heavily hunted populations.

We used a quadratic regression for the approximation of the association between maximum lifespan and age at reproductive senescence.

## Results and Discussion

**Figure 1:**
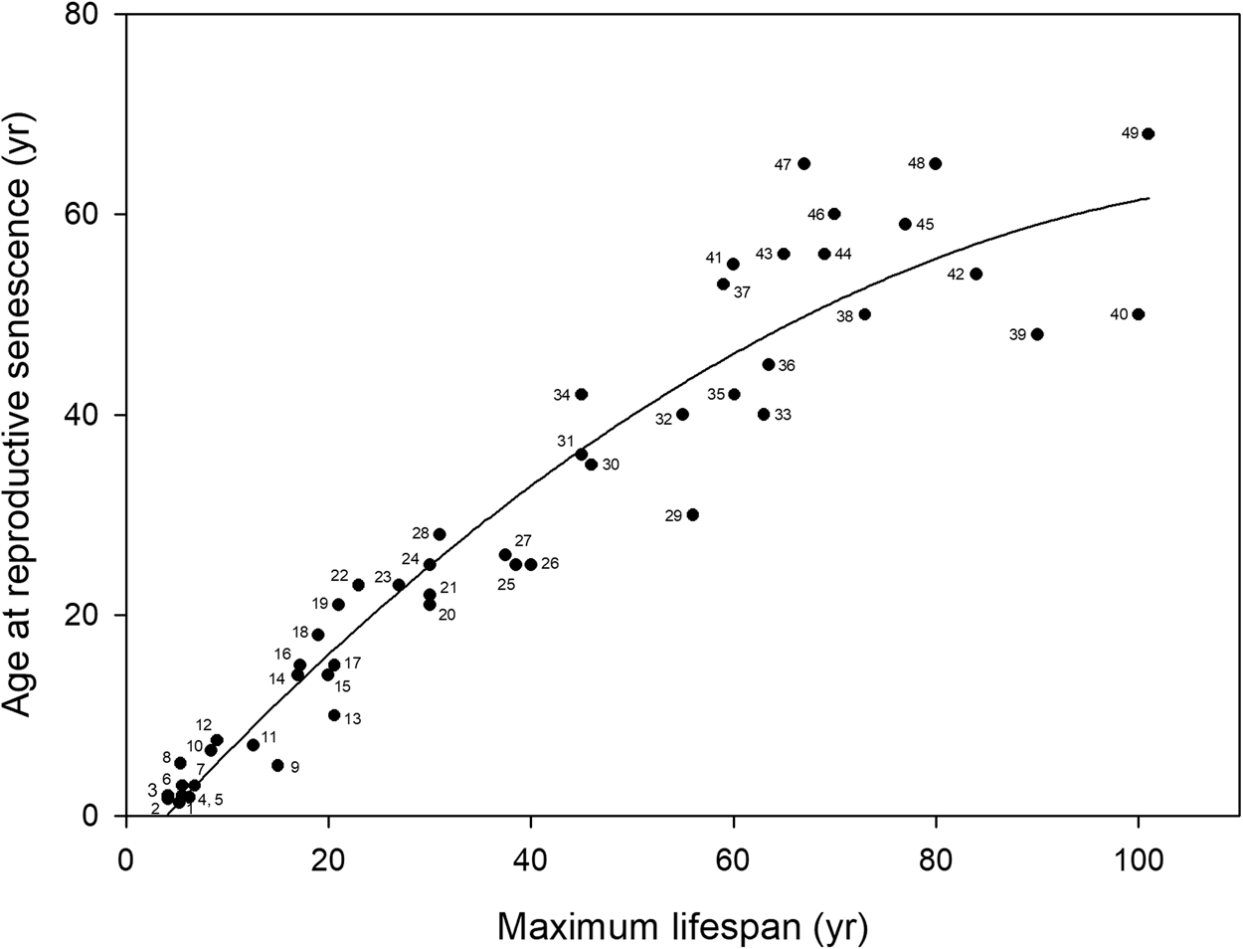
Age at reproductive senescence (yr) versus maximum lifespan (yr) in 49 mammal species (excluding baleen whales). Data points fitted by a quadratic regression (line; RS = −4.4535^*^+1.1168 LS^***^ − 0.0046 LS^2***^; adjusted R^2^ = 0.9201; *p < 0.05, ***p < 0.001). Labels (sources): 1 *Cricetulus barabensis* (AnAge), 2 *Mus musculus*^29^, 3 *Rattus norvegicus*^29^, 4 *Phascogale tapoatafa* (AnAge), 5 *Meriones unguiculatus* (AnAge), 6 *Onychomys leocogaster* (AnAge), 7 *Dasyurus maculatus* (AnAge), 8 *Peromyscus truei* (AnAge), 9 *Oryctolagus cuniculus*^36^, 10 *Ochrotomy nuttalli* (AnAge), 11 *Hypogeomys antimena* (AnAge), 12 *Spermophilus comlumbianus*^36^, 13 *Canis lupus* (AnAge), 14 *Panthera leo*^36^, 15 *Felis catus*^36^, 16 *Chinchilla lanigera* (AnAge), 17 *Cryptomys anselli* (AnAge), 18 *Ovis canadensis*^36^, 19 *Propithecus diadema* (AnAge), 20 *Macaca nemestrina*^36^, 21 *Ursus maritimus*^36^, 22 *Odocoileus virginianus* (AnAge), 23 *Papio cynocephalus*^36^, 24 *Bos taurus*^36^, 25 *Macaca fuscata* (AnAge), 26 *Macaca mulatta* (AnAge^36^), 27 *Papio hamadryas* (AnAge), 28 *Propithecus verreauxi* (AnAge), 29 *Pusa sibirica* (AnAge), 30 *Stenella attenuata* (AnAge), 31 *Ceratotherium simum* (AnAge), 32 *Pan paniscus* (AnAge^59^), 33 *Globicephala macrorhynchus* (AnAge), 34 *Equus caballus*^29^, 35 *Gorilla gorilla* (AnAge^60^), 36 *Pseudorca crassidens*^61^, 37 *Pongo pygmaeus* (AnAge), 38 *Dugong dugon* (AnAge^62^), 39 *Orcinus orca* (AnAge), 40 *Homo sapiens* (AnAge) (note: according to AnAge, 100 yr is used as maximum lifespan), 41 *Globicephala melas* (AnAge), 42 *Berardius bairdii* (AnAge^63^), 43 *Pan troglodytes* (AnAge^36^), 44 *Trichechus manatus* (AnAge^37^) (note: age at reproductive senescence is age at which 95% of population fecundity is reached), 45 *Physeter macrocephalus* (AnAge^63^), 46 *Loxodonta africana* (AnAge), 47 *Eubalaena glacialis* (AnAge), 48 *Elephas maximus*^64^, 49 *Monodon monoceros*^65^.

Unsurprisingly, we find a strong positive association between maximum lifespan and age at reproductive senescence. But this positive association levels off in long-lived species (Fig. 1): both, the distribution of the data points and the lower AIC for the quadratic model, suggest a non-linear association (Table 1). Thus, in long-lived species, the age at reproductive senescence appears to have an upper limit, providing evidence for a maximum shelf-life of oocytes.

**Table 1:** Parameters and Akaike information criterion of the linear and the quadratic model fitted to the data of Fig. 1.

	Intercept	x	x^2^	adjusted R^2^	AIC
linear model	1.4438	0.6957^***^		0.8948	330.0762
quadratic model	−4.4535*	1.1168^***^	−0.0046^***^	0.9201	317.5733

*p < 0.05, ***p < 0.001.

Exceptions seem to be the baleen whales (mysticetes) (data not shown) as there is little indication for reproductive senescence in any of the baleen whales so far^41,42^. We can only speculate why baleen whales do not fit in the trend. Possibly, in baleen whales, selection pressure to extend the reproductive lifespan was particularly high, so that they may have found a way to overcome this putative constraint. A reason for a strong selection pressure to extend reproductive lifespan could be the fact that as an adaptation to water and a foraging strategy as obligate filter feeders, baleen whales evolved very large body sizes together with very long lifespans of up to 200 years. Indeed, out of the 5 longest living mammal species, four are baleen whales and the remaining one is *Homo sapiens* (AnAge).

Yet, apart from the mysticetes, our finding supports the notion that the length of time oocytes can remain viable may be the limiting factor of reproductive lifespan^34,35^. This assumption implies that there should be a process during follicular development that cannot be extended for an unlimited period of time. As oocytes remain inactive in an arrested phase of meiosis from their production *in utero* until they either undergo atresia or undergo ovulation, we suggest that meiotic arrest may be a prime candidate for such a process. Indeed, the frequency of chromosomal non-disjunctions increases with advancing age of oocytes^43,44^. Also chromosomal errors increase as the state of meiotic arrest is prolonged^45–47^.

In fetal ovaries, oogonia enter meiosis I to become primary oocytes that halt their development at prophase of meiosis I, the dictyate stage. This arrest of meiosis I continues until cycling, when a few primary oocytes are recruited during each cycle and one oocyte per ovulatory cohort matures to ovulation, the others providing hormonal support. During this maturation, the oocyte finishes meiosis I but meiosis II is again arrested – at metaphase - and only terminates after successful fertilization^48^.

In humans, dependent upon the woman’s age, meiotic arrest of meiosis I lasts roughly between 12 and 52 years. Consistent with the view that the duration of meiotic arrest may be a crucial factor, most chromosomal abnormalities are of maternal origin and mostly arise from chromosomal non-disjunction of meiosis I^49–51^, leading to either monosomy or trisomy. Moreover, the frequency of aneuploidity dramatically increases with advancing maternal age^52^. Although there is a moderate increase in frequency of all trisomies occurring in clinically recognized pregnancies in women younger than approximately 33 years, from about 2% in women younger than 25 years to about 6% in women aged 33 years^49,53^, after the age of about 33 years, trisomy risk increases exponentially to about 30% in women 40 years of age and 35% in women aged 42 years and older^49,53,54^. Even more, as all autosomal monosomies and most trisomies lead to fetal death^55^, in abortuses of women over 40 years of age, chromosomal abnormalities are found in 85% of cases^56^.

We assume that the most likely cause of this age related increase of meiotic non-disjunctions may be the deterioration of cohesion of sister chromatids during meiosis I^57^. Cohesion is clearly a good candidate for a process that deteriorates with increasing period of time. Moreover, this view is supported by findings of deterioration of cohesion in aged mice and in humans^57,58^.

In conclusion, we find that the positive association of reproductive and somatic senescence levels off in long-lived mammals, indicating an upper limit of reproductive lifespan. Only the baleen whales appear to be an exception. We suggest that this failure to further postpone reproductive senescence commensurate with increasing lifespan may be due to an inability to extend meiotic arrest for an unlimited period of time. We therefore propose that menopause may be an implication of the long duration of meiotic arrest in oocytes caused by semelgametogenesis together with long lifespan.

## Data Availability

All data used in the analysis are obtained from the cited sources.
